# Application of Potato Peels as an Unconventional Sorbent for the Removal of Anionic and Cationic Dyes from Aqueous Solutions

**DOI:** 10.3390/ma19010185

**Published:** 2026-01-04

**Authors:** Tomasz Jóźwiak, Urszula Filipkowska, Anna Nowicka, Jarosław Kaźmierczak

**Affiliations:** Department of Environmental Engineering, University of Warmia and Mazury in Olsztyn, Warszawska St. 117a, 10-957 Olsztyn, Poland; urszula.filipkowska@uwm.edu.pl (U.F.); anna.grala@uwm.edu.pl (A.N.); krown@interia.pl (J.K.)

**Keywords:** potato peels, biomass, unconventional sorbent, sorption, dyes, RY84, RB5, BR46, BV10

## Abstract

The aim of this study was to investigate the sorption efficiency of anionic dyes—Reactive Yellow 84 (RY84) and Reactive Black 5 (RB5)—and cationic dyes—Red 46 (BR46) and Basic Violet 10 (BV10)—onto potato peels (*Solanum tuberosum* L.). The research scope included characterization of the sorbent material (pH_PZC_, FTIR), the effect of pH on dye sorption efficiency, kinetics (pseudo-first-order and pseudo-second-order models, intraparticle diffusion model), and studies on the sorbent’s maximum sorption capacity (Langmuir 1 and 2, and Freundlich isotherms). The point of zero charge (pH_PZC_) for potato peels was determined to be pH_PZC_ = 6.43, indicating a slightly acidic character of the material. The sorption efficiency for RB5, RY84, and BV10 was highest at pH 2, while the efficiency for BR46 was highest at pH 6. The time required to reach sorption equilibrium on the tested sorbent increased with the initial dye concentration and ranged from 180 to 270 min for RB5, RY84, and BV10, and from 45 to 210 min for BR46. The maximum sorption capacity of this material was found to be 20.85 ± 0.33 mg/g and 21.63 ± 0.34 mg/g for RB5 and RY84, respectively, and 10.28 ± 0.24 mg/g and 27.15 ± 0.87 mg/g for BV10 and BR46, respectively.

## 1. Introduction

Dyes are chemical compounds characterized by their ability to absorb electromagnetic radiation in the 380–780 nm range, which consequently imparts their color. Primarily utilized for industrial coloration, these compounds are produced at a rate of approximately 1.1 million tons annually, with over 10,000 distinct types currently identified [1]. A significant majority of this production—estimated at 75%—is utilized specifically for textile manufacturing processes [2]. Unfortunately, during the dyeing process, a significant fraction of these compounds fails to bind permanently to the material’s surface and is released into post-production wastewater. The highest dye losses, reaching up to 50%, are specifically observed with anionic reactive dyes [3]. These intensely colored industrial effluents are recognized as a serious environmental threat to natural ecosystems.

The infiltration of colored effluent streams into natural water systems can lead to severe ecological damage. In water bodies, the presence of dyes restricts the availability of sunlight for aquatic flora, thereby decreasing the overall photosynthetic rate of the ecosystem [4]. Due to the reactivity of many dyes with dissolved oxygen and the reduced oxygen output from autotrophs, the system’s dissolved oxygen levels may drop to critical levels, inducing anaerobic conditions [5]. Adding to this environmental burden, some dye compounds exhibit high toxicity toward aquatic life [6]. As a result, the presence of these pigments plays a substantial role in the ecological deterioration of natural water bodies [7]. To reduce the risk of water bodies being contaminated by dyes, the best available technologies for colored wastewater decolorization should be used.

Most dyes currently produced are classified as poorly biodegradable substances [8]. As a result, traditional biological methods used for treating colored wastewater are generally inefficient [9]. Many scientists agree that sorption is among the most effective strategies for wastewater decolorization [10]. This process involves the binding of one substance (the sorbate) by the sorbent. The overall efficiency of the sorption process depends on operational parameters, including pH and temperature, as well as the specific type of sorbent [11]. Activated carbon remains the predominant sorbent for dye removal [12]. This material is produced through the carbonization and subsequent activation of organic feedstocks rich in carbon, such as hard coal, wood, nut shells, and coal tar pitch [13]. Activated carbon exhibits a highly developed specific surface area, typically exceeding 500 m^2^/g, which allows it to bind most dyes present in wastewater [14]. Furthermore, spent activated carbon can be regenerated, although this process usually results in a sorbent mass loss of 10 to 15% [15]. A drawback of activated carbon is its relatively high price, mainly due to high production costs [16]. A frequently used alternative to activated carbon is mineral materials belonging to the aluminosilicates (zeolites, bentonites, montmorillonites). They are cheaper than activated carbon, but their lower specific surface area results in poorer sorption capacity [17]. Moreover, they are not as versatile as activated carbon and are ineffective against certain types of dyes, such as anionic dyes [18].

Researchers are currently seeking inexpensive and efficient sorption materials to serve as alternatives to conventional sorbents. To reduce costs, raw materials for producing unconventional sorbents are increasingly sourced from industrial waste [19]. There is particular interest in obtaining sorption materials from waste products of the agri-food industry [20]. These raw materials can be classified as either animal or plant in origin.

Tested “waste sorbents” of animal origin include keratin materials (bird feathers [21], fish scales [22]), chitin materials (crustacean shells [23], chitosan [24]), and calcium carbonate-based materials (snail shells [25], eggshells [26]). A general drawback of keratin materials is their relatively small surface area, which limits their sorption capacity. Calcium carbonate-based sorbents are useful for removing anionic dyes but are susceptible to dissolution in acidic environments [27]. Chitin and chitosan sorbents yield very good results and can have better sorption capacities than activated carbon-based materials [28]. However, these materials are less available in countries where marine crustaceans are not harvested.

A significantly larger group of “waste sorbents” consists of plant-based materials. These are generally cheaper and even more readily available than animal-based waste. They are mostly plant-derived waste, such as leaves and stems [29], seed hulls [30], nut shells [31], and fruit peels [32]. Plant biosorbents can bind anionic dyes, though they are usually much more efficient against cationic dyes [33]. The sorption characteristics observed in plant biomass are intrinsically linked to its fundamental composition, primarily its content of polysaccharides (including cellulose, hemicellulose, and starch), lignin, and various proteins. Another example of waste plant biomass that, due to its composition, could serve as a plant sorbent is potato peels.

Potatoes are among the most popular and cheapest vegetables worldwide [34]. They form the dietary foundation for billions of people. Their annual production reaches 383 million tons (2023) [35]. Before consumption, potatoes are most often peeled. The peeling method used determines the yield of the resulting biomass, with potato peels typically accounting for 15% to 40% of the initial potato mass [36]. Since potatoes are consumed in most countries globally, especially in Europe and Asia [37], potato peels can be considered a readily available material in very large quantities. In the context of food industry waste, potato peels are often utilized in agriculture, for example, as a feed additive, a component of fertilizers [38] or compost [39], or as a substrate for biogas production in biogas plants [40]. However, in households, potato peels are most often treated as waste and discarded as garbage. The high polysaccharide content in potato peels, exceeding 60% of their dry mass [41], may indicate their high potential as a dye-binding material.

This research seeks to quantify the sorption performance of potato peels (*Solanum tuberosum* L.) toward a selection of widely used industrial dyes, including the anionic dyes (RY84, BR5) and the cationic dyes (BR46, BV10).

## 2. Materials and Methods

### 2.1. Potato Peels

The peels were obtained from “BRYZA” variety potatoes (*Solanum tuberosum* L.) harvested in early September 2024 from a farm in the Olsztynek commune, Warmian-Masurian Voivodeship, Poland. After harvesting, the potatoes were stored for six months in canvas bags at 7 °C and 75% relative humidity. Before peeling, the potatoes were repeatedly washed with deionized water until the removal of all terrestrial contaminants was achieved. The peels were then collected for experimentation in early March 2025 using a standard kitchen potato peeler. The resulting biomass had a peel thickness ranging from 0.5 to 1.0 mm. The detailed chemical composition of the potato peels is provided in Table 1.

### 2.2. Dyes

Commercial-grade dyes were purchased from Boruta-Zachem SA (Zgierz, Poland).

**Figure 1:**
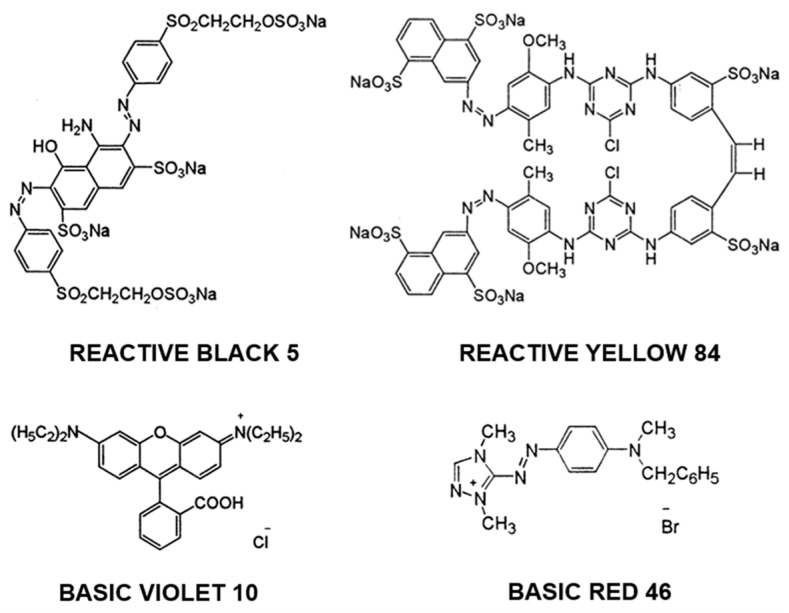
Chemical structure of dyes used in the study.

The study included two anionic dyes (RY84, RB5) and two cationic dyes (BR46, BV10) (Figure 1). Specific characteristics of each dye utilized during the experiments are compiled in Table 2.

### 2.3. Chemical Reagents

The study utilized the following set of chemical reagents:

- NaOH (sodium hydroxide) > 99.9%—(pH correction of dye solutions);
- HCl (hydrochloric acid)—37%—(pH correction of dye solutions)

All chemical substances used during the experiments, characterized by analytical grade purity (p.a.) or superior, were supplied by POCH S.A. (Gliwice, Poland).

### 2.4. Laboratory Equipment

The experimental work was supported by a range of specialized laboratory instruments. Biomass preparation involved the use of an A 11 basic grinder (IKA-Werke GmbH & Co. KG, Staufen im Breisgau, Germany) to process the potato peels. For monitoring and regulating the acidity of the samples, a HI 221 pH-meter (Hanna Instruments, Smithfield, RI, USA) was employed. Concentration levels of the dyes were quantified via spectrophotometry using a UV-3100 PC unit (VWR International LLC., Mississauga, ON, Canada). Furthermore, the surface characteristics of the sorbent were analyzed through an FT/IR-4700LE spectrometer (JASCO International, Tokyo, Japan) equipped with an ATR module. The sorption experiments were carried out using an SK-71 laboratory shaker (JEIO TECH, Daejeon, Republic of Korea) to ensure proper mixing.

### 2.5. Sorbent Preparation—Potato Peels (PP)

Initially, the gathered potato biomass underwent fragmentation in a mechanical laboratory mill. To ensure uniformity, the processed peels were classified by size using a sieving system with 2 mm and 3 mm apertures. Only the fraction retained between these limits (2–3 mm) was selected for further use. This material was subsequently purified through intensive rinsing with deionized water, a process continued until the wash water reached total clarity. The washed peel fraction was then spread on laboratory blotting paper for 5 min to remove water between the sorbent particles (the peels were not dried). The potato peel biomass (PP) obtained in this way, with a dry matter content of 22.3–22.7%, was then ready for testing. The PP was always prepared immediately before each research series.

### 2.6. FTIR Analysis of the PP

To identify the surface functional groups of the potato peels (PP), FTIR analysis was conducted via a FT/IR-4700LE instrument (JASCO International, Tokyo, Japan). The study utilized the Attenuated Total Reflectance (ATR) mode, featuring a diamond crystal for single-reflection measurements. The infrared spectra were recorded within a wide frequency range (4000–400 cm^−1^). To minimize background noise and achieve high spectral precision, each final result was an average of 64 individual scans, maintaining a resolution of 1 cm^−1^.

### 2.7. Impact of Initial Solution pH on the Sorptive Removal of Dyes by PP

Dry PP samples (2.50 g each) were measured into 500 mL conical vessels for the subsequent batch experiments. All weighing operations were conducted using an analytical balance to maintain accuracy. Working dye solutions (250 mL) at a concentration of 50 mg/L, with initial pH values ranging from 2 to 11, were then added to these flasks. The flasks were placed on a laboratory shaker (120 rpm, oscillation amplitude 30 mm) for 120 min. Following the equilibration time, 10 mL aliquots were collected from each flask using a digital pipette and transferred into polyethylene containers. These samples were then analyzed via spectrophotometry to quantify the concentration of the remaining dye. Furthermore, the equilibrium pH of each system was recorded promptly at the end of the process. Data regarding the calculated ionic strength for the various pH conditions are summarized in Table 3.

### 2.8. Kinetic Studies of Dye Sorption onto PP

The kinetic experiments began by weighing 20.00 g (dry mass) of the PP sorbent into 2000 mL laboratory beakers. Then, 2000 mL of dye solutions were added, using concentrations of 50, 200, and 500 mg/L for the dyes RY84, RB5 and BR46, and 10, 50, and 200 mg/L for the dye BV10. All solutions were prepared at the optimal sorption pH, as determined in the studies detailed in Section 2.7. The difference in concentration ranges was necessary due to the significantly lower sorption efficacy observed for BV10 on the PP biomass. A Teflon-coated magnetic stirring bar (PTFE, 80 × 10 mm) was placed in each beaker. The mixture was stirred at 200 rpm using magnetic bars (dimensions: 80 × 10 mm). Kinetic data collection involved periodic withdrawal of 5 mL solution portions at 0, 10, 20, 30, 45, 60, 90, 120, 150, 180, 210, 240, 270, and 300 min. These samples were collected with an automatic pipette and secured in test tubes for further analytical procedures.

### 2.9. Determination of the Maximum Sorption Capacity of PP for Dyes

Equilibrium studies were performed by introducing 2.50 g of dry PP sorbent into a series of 500 mL Erlenmeyer flasks. These vessels were filled with 250 mL of dye solutions, where the initial concentrations were varied between 10 and 500 mg/L. To ensure maximum efficiency, the pH of each mixture was adjusted to the optimal value identified in Section 2.7. The system was then agitated using a laboratory shaker (120 rpm, 30 mm amplitude) until the equilibrium state, previously determined in Section 2.8, was achieved. Subsequently, 10 mL aliquots were extracted via a digital pipette to assess the concentration of the dye remaining in the liquid phase.

*Comments on Section 2.7, Section 2.8 and Section 2.9*

- PP (potato peels) was tested against four dyes: RB5, RY84, BV10, and BR46.
- Dye stock and working solutions were prepared exclusively with high-purity deionized water.
- All batch experiments were conducted thrice (in triplicate) to ensure the statistical reliability of the collected data.
- The PP dosage was kept constant at 10.00 g d.m./L (grams dry matter per liter) in all experiments.
- The research used PP in its fresh, not dried, form.
- The PP portions were accurately weighed (to ±0.001 g) using a precision balance.
- Continuous agitation, provided by either the orbital shaker or the magnetic stirring units, facilitated the complete and consistent suspension of the biomass across the entire reactive volume.
- Dye concentrations were quantified by spectrophotometry using a UV-VIS spectrophotometer with a 10 mm optical path length cuvette.
- Standard curves permitted the determination of RB5, RY84, and BR46 levels within a 50 mg/L limit, whereas BV10 concentrations were measured using a 0–10 mg/L calibration scale. Curves were prepared at λ_max_ (values given in Table 2, Section 2.2). Solutions exceeding the linearity range of the calibration curves were diluted with deionized water before measurement.
- The laboratory air temperature was maintained at a stable 25 °C throughout the analyses.

### 2.10. Calculations

The quantity of dye captured by the PP sorbent was determined according to the relationship expressed in Equation (1):

(1) $$Q_{S}=(C_{0}-C_{S})\times \frac{V}{m}$$

Q_S_—amount of dye adsorbed [mg/g]
C_0_—initial dye concentration in solution [mg/L]
C_S_—dye concentration remaining in solution after sorption [mg/L]
V—volume of the dye solution utilized [L]
m—mass of the dry sorbent used [g]

The kinetic mechanism governing the dye sorption process onto PP was described by applying the pseudo-first-order model (2), the pseudo-second-order model (3), and the intraparticle diffusion model (4).

(2) $$q=q_{e}\times (1-e^{\left(-k_{1}\times t\right)})$$

(3) $$q=\frac{\left(k_{2}\times {q_{e}}^{2}\times t\right)}{\left(1+k_{2}\times q_{e}\times t\right)}$$

(4) $$q=k_{id}\times t^{0.5}$$

q—instantaneous amount of dye adsorbed at time [mg/g].
q_e_—the amount of dye sorbed at equilibrium [mg/g].
t—sorption contact time [min].
k_1_—the rate constant of the pseudo-first-order kinetic model [1/min].
k_2_—the rate constant of the pseudo-second-order kinetic model [g/(mg*min)].
k_id_—the intraparticle diffusion rate constant [mg/(g*min^0.5^)].

Experimental data derived from studies investigating the maximum sorption capacity of PP for the dyes were modeled using the following equations: Langmuir 1 (single-site) (5), Langmuir 2 (double-site) (6), and Freundlich (7).

(5) $$Q=\frac{\left(Q_{max}\times K_{C}\times C\right)}{\left(1+K_{C}\times C\right)}$$

(6) $$Q=\frac{\left(b_{1}\times K_{1}\times C\right)}{\left(1+K_{1}\times C\right)}+\frac{\left(b_{2}\times K_{2}\times C\right)}{\left(1+K_{2}\times C\right)}$$

(7) $$Q=K\times C^{\frac{1}{n}}$$

Q—amount of dye adsorbed at equilibrium [mg/g].
Q_max_—maximum monolayer sorption capacity in the Langmuir model [mg/g].
b_1_—the theoretical maximum loading of the high-energy (Type I) active centers [mg/g].
b_2_—the theoretical maximum loading of the high-energy (Type II) active centers [mg/g].
K_C_—Langmuir adsorption constant [L/mg].
K_1_, K_2_—adsorption constants for type I and type II sites in the Langmuir 2 model [L/mg].
K—Freundlich equilibrium constant (sorption capacity indicator).
n—Freundlich adsorption intensity indicator.
C—the equilibrium dye concentration in the aqueous phase [mg/L].

The ionic strength of the dye solutions was calculated from Formula (8)

(8) $$I=\frac{1}{2}\sum_{i=1}^{n}c_{i}\times z_{i}^{2}$$

I—ionic strength of the solution [mol/L].
c_i_—molar concentration of the i-th ion [mol/L].
z_i_—charge number of the i-th ion.
∑—summation over all ions in the solution.

## 3. Results and Discussion

### 3.1. Characterization of the PP

#### 3.1.1. FTIR Analysis

**Figure 2:**
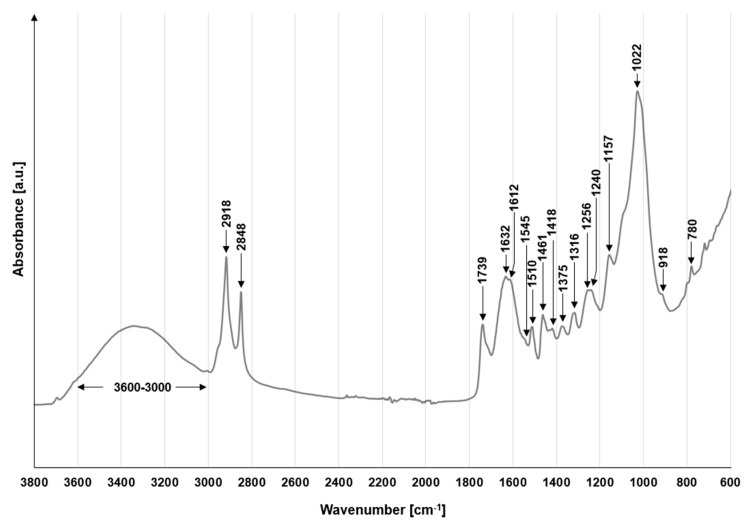
FTIR spectra for PP.

The FTIR profile of potato peels (PP) is typical of plant biomass (Figure 2). The spectral range from 1200 to 800 cm^−1^ is highly characteristic for carbohydrate structures and is frequently referred to as the “fingerprint” zone for various polysaccharides [45]. The weak peak at 1157 cm^−1^ indicates the asymmetric stretching of the C–O–C alpha-1,4-glycosidic linkage, characteristic of starch [46]. The presence of starch in the material is also suggested by the peak at 1022 cm^−1^, which is attributed to C–O stretching vibrations and C–O–H deformation vibrations [47]. The high intensity of the 1022 cm^−1^ peak, along with the lack of visibility of the 1045 cm^−1^ peak, also suggests an amorphous (non-crystalline) structure of the starch in the tested sorbent [48]. In the region spanning 1200–800 cm^−1^, the peak at 918 cm^−1^ originates from internal stretching within the pyranose rings. This vibration arises from the C–C and C–O linkages that form the backbone of numerous polysaccharides [49].

The signals at 1510 cm^−1^ and 1612 cm^−1^ are associated with C=C stretching vibrations in aromatic rings and indicate the presence of lignin in the sorbent structure [50]. The peak at 1545 cm^−1^, related to N-H bending and C-N stretching vibrations, indicates the presence of proteins in the material [51].

In addition to the peaks mentioned above, the PP spectrum contains many non-specific peaks that may result from various compounds. The broad band between 3600 cm^−1^ and 3000 cm^−1^ is typically attributed to O-H bond stretching, characteristic of -OH groups found in polysaccharides (cellulose, starch), lignin, and water [45,52]. The presence of aliphatic structures within the PP matrix is confirmed by the dual signals at 2918 cm^−1^ and 2848 cm^−1^. These bands are attributed to the asymmetric and symmetric stretching modes of methylene (-CH_2_-) bridges. Such features are typical for a variety of biopolymers, including lipid-derived chains, lignin frameworks, or the carbohydrate backbones of various polysaccharides [50,53,54]. The peaks at 1739 cm^−1^ and 1632 cm^−1^ can be attributed to stretching vibrations of the carbonyl bond (C=O) of ester and acidic functional groups, which may be related to hemicellulose, lignin, and pectin in the tested material [55,56].

The signals at 1461 cm^−1^ and 1375 cm^−1^ correspond to asymmetric and symmetric bending vibrations of the C-H bonds of methyl (-CH_3_) and methylene (=CH_2_) groups in polysaccharides, lignins, and lipids [57]. The peak at 1418 cm^−1^ may indicate C-H bending vibrations of aliphatic chains in polysaccharides and lignins [57]. The absorption maximum at 1316 cm^−1^ can be assigned to the C1-O stretching vibration of the syringyl unit of lignin and the C-H deformation vibrations in the glucosyl rings of polysaccharides [58]. The absorbance features recorded at 1256 cm^−1^ and 1240 cm^−1^ are likely associated with the valence oscillations of ether (-C-O-C-) and ester (-C(O)=O-) linkages. These signals point to the presence of lignin-based structures and various polyphenolic constituents within the PP biomass [59]. A small peak at 780 cm^−1^ corresponds to out-of-plane C-H deformation vibrations in aromatic rings, which may belong to lignins and phenolic acids present in PP [60].

#### 3.1.2. Determination of the Sorbent pH_PZC_

**Figure 3:**
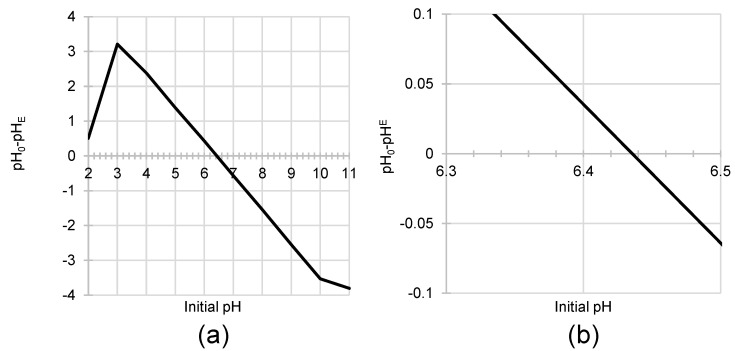
(**a**,**b**) pH_PZC_ of PP determined by the “drift” method. Temp. 25 °C.

As shown in Figure 3, the point of zero charge for the tested PP material was determined to be 6.43. This value was obtained using the potentiometric drift procedure. This indicates the sorbent is slightly acidic [61], likely because the chemical structure of potato peels contains more acidic functional groups (e.g., carboxyl, sulfonic, and phosphate groups) than basic groups (e.g., amine groups). The acidic character of the material may also suggest higher efficiency in removing cationic dyes compared to anionic dyes.

### 3.2. Effect of pH on Dye Sorption by PP

**Figure 4:**
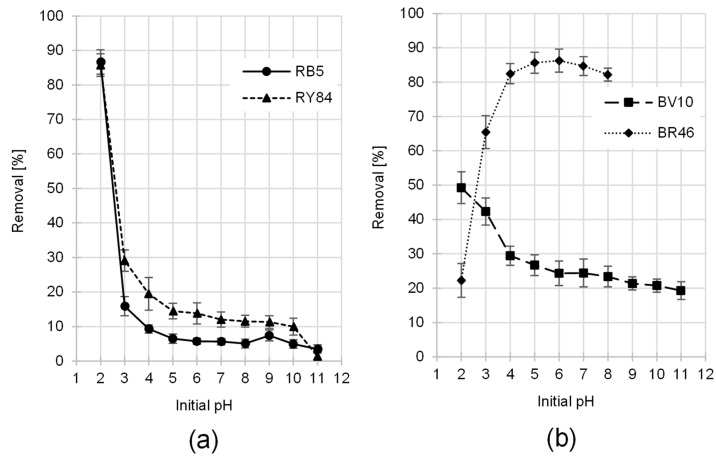
The influence of pH on the adsorption performance of dyes: (**a**) RB5, RY84 (**b**) BV10, BR46 PP (average + standard deviation). Initial conc. of dyes = 50 mg/L. Temp. 25 °C.

The uptake of anionic reactive dyes by the PP biomass was found to be highly pH-dependent, with the greatest removal efficiency recorded at pH 2. A continuous downward trend in sorption performance was observed as the alkalinity increased, with the lowest adsorption levels occurring at pH 12. (Figure 4a). The largest decrease in the binding efficiency of RB5 and RY84 occurred between pH 2 and 3, while from pH 5 to 10, the sorption effectiveness remained similarly low. Interestingly, the sorption performance for RB5 exhibited a minor enhancement when the system reached pH 9, deviating slightly from the general downward trend.

A similar effect of pH on the sorption effectiveness of anionic dyes, characterized by high binding intensity at low pH, has also been observed in studies on the sorption of RB5 onto cotton seed husks [62], and commercial activated carbon [28]. Comparable results were also reported in studies on RY84 sorption onto cotton fibers [63] and sunflower seeds [64].

The strong binding efficiency observed for the anionic dyes under low pH conditions was primarily attributed to the PP surface acquiring a net positive charge. This positive charge is induced by the excess hydronium ions (H_3_O^+^) present in the acidic environment. The charge acquisition on the sorbent likely results from a combination of protonation of the PP’s native functional groups and the physical adsorption of hydronium ions. The functional groups primarily undergoing protonation were the amine groups from proteins [65] and, to a lesser extent, the hydroxyl groups from polysaccharides (starch, cellulose, hemicellulose) and lignin [66].

-NH_2_ + H_3_O^+^ → -NH_3_^+^ + H_2_O (protonation effective even at pH < 9)

-OH + H_3_O^+^ → -OH_2_^+^ + H_2_O (low protonation efficiency even at pH < 3)

The deionization of carboxyl groups from proteins or partially degraded lignin also contributed to the overall positive charge on the PP surface:

-COO^−^ + H_3_O^+^ → -COOH + H_2_O (deionization through proton attachment at pH < 3)

The positive electrification of the PP sorbent at pH values below 4 was largely driven by the incorporation of hydronium species. These cations became anchored to the structure by forming intermolecular hydrogen bridges with the -OH functionalities of the potato peels, as illustrated by the following interaction:

-OH + H_3_O^+^ → -OH⋯H-OH_2_^+^

“⋯” denotes the formation of a hydrogen bond [67].

The sulfonic groups of dyes RB5 and RY84 existed in their ionized form across the entire tested pH range (2–11), meaning these dyes always carried a negative charge [68]:

-SO_3_H + H_2_O → -SO_3_^−^ + H_3_O^+^

Under acidic conditions (low pH), the pronounced positive charge on the PP surface resulted in strong electrostatic attraction for the dye anions, substantially enhancing their sorption efficiency. Conversely, as the pH increased, the concentration of H_3_O^+^ ions in the solution decreased. This reduction led to decreased effectiveness in both the protonation of the sorbent’s functional groups and the physical adsorption of H_3_O^+^ ions. The resulting decrease in the positive charge of the sorbent led to progressively lower binding effectiveness of RB5 and RY84 onto PP. The greatest drop in the positive charge of the sorbent surface, observed when the pH increased from 2 to 3, resulted primarily from the lower efficiency of hydronium ion adsorption onto PP (caused by a tenfold lower H_3_O^+^ concentration). Furthermore, at pH 3, protonation of hydroxyl groups practically ceased, while most carboxyl groups became deprotonated. This explains the sudden decrease in the sorption effectiveness of RY84 and RB5 onto PP observed in the pH 2–3. Within the broad pH interval of 4 to 10, the binding of anionic dye molecules was likely mediated by the trace amounts of amino functionalities still available on the PP surface. In an alkaline environment with an excess of hydroxide ions, the PP surface acquired an overall negative charge [69]. This was mainly due to the deprotonation of -COOH and -OH functional groups:

-OH + OH^−^ → -O^−^ + H_2_O (effective deprotonation at pH > 11)

-COOH + OH^−^ → -COO^−^ + H_2_O (effective deprotonation already at pH ≥ 3)

The negatively charged sorbent surface electrostatically repelled the anionic dyes, further hindering their binding [70]. This explains the very low sorption effectiveness of RB5 and RY84 onto PP at pH 11 (Figure 4a).

The minor enhancement of RB5 adsorption observed near pH 9 (Figure 4a) is likely a consequence of the intrinsic amine group present in the dye molecule. At this pH, the potato peel surface had probably already acquired an overall net negative charge. At the same time, a significant fraction of the RB5 molecules still carried a localized positive charge from the protonated amine group. The enhanced uptake observed can be attributed to the specific coulombic interaction occurring between the anionic sites of the PP surface and the cationic form of the dye’s amine moiety. At pH > 9 most RB5 molecules are assumed to have their amine groups in the unionized form, resulting in weaker interactions between the sorbent and sorbate and a measurable decrease in dye sorption efficiency. This phenomenon was not observed for RY84, which is consistent with the absence of amine functional groups in its structure.

The adsorption profile of BR46 onto the PP sorbent showed an upward trend as the solution became less acidic, with peak efficiency achieved at pH 6 (Figure 4b). A further increase in alkalinity led to a slight reduction in the sorption capacity. Notably, the steepest gain in efficiency was observed during the initial pH shift from 2 to 4.

Data for the pH range of 9–11 were excluded from Figure 4b because spontaneous blanching of BR46 solutions was observed at pH > 8 (i.e., without contact with the sorbent). The loss of color in BR46 under alkaline conditions is attributed to a reaction between hydroxide ions (OH^−^) and the electrophilic triazolium group within the BR46 chromophore system. If included, this chemical loss of color intensity in the pH 9–11 range could have been misinterpreted as a genuine sorption effect.

The observed pH-dependence of BR46 uptake, specifically the transition from minimal binding in acidic media to peak efficiency at pH 6, is consistent with findings for other waste-based sorbents, such as rapeseed husks [71], and spent green tea leaves [72]. These studies also noted the spontaneous decolorization of BR46 solutions in alkaline environments and chose not to include results for the sorption of this dye at high pH.

The low sorption effectiveness of BR46 onto the tested sorbent at low pH resulted from the basic (cationic) nature of the dye. At pH 2, the high density of positive charges on the PP surface created a coulombic repulsion against the BR46 cations, thereby suppressing their adsorption capacity. As the system’s pH increased above 2, the -OH groups, which make up the majority of the sorbent’s functional groups, remained un-ionized. Furthermore, as previously mentioned, at pH > 2, the efficiency of hydronium ion adsorption onto PP significantly decreased, reducing the overall positive charge on the PP surface. Additionally, at pH > 3, carboxyl groups underwent deprotonation, enabling effective electrostatic binding of BR46 to the PP surface. This explains the sudden increase in the sorption effectiveness of Basic Red 46 onto the tested sorbent observed in the pH 2–4 range.

In contrast to BR46, the removal efficiency of the cationic dye BV10 showed a trend more characteristic of anionic species. Its maximum uptake was recorded at pH 2 and steadily diminished as the pH rose, hitting a nadir at pH 11 (Figure 4b). A characteristic atypical for a cationic dye is that Basic Violet 10 possesses a carboxyl functional group in its structure. The -COOH group easily ionizes in an aqueous environment. Consequently, a significant portion of the BV10 particles, despite their overall cationic character, carry local negative charges. The deprotonated carboxyl groups can interact electrostatically with the positively charged PP surface, allowing BV10, under certain conditions, to “behave” similarly to anionic dyes.

A comparable trend regarding the influence of pH on BV10 sorption effectiveness has also been reported in studies investigating the adsorption of this dye onto spent coffee grounds and green tea grounds [72].

Beyond pure electrostatic forces, the sequestration of all studied dyes likely involved the development of hydrogen bridges. These interactions occur between the hydroxyl functionalities of the PP matrix and the tertiary amino moieties present in the dye molecules. Furthermore, π−π stacking between the lignin-derived aromatic frameworks and the dye’s benzene rings could significantly contribute to the overall binding energy.

The ionic strength of the dye solutions used in the studies depended on the pH and the type of dye, and its value ranged from 1.04 × 10^−4^ mol/L at pH 7 (BR46 solution, 50 mg/L) to 1.06 × 10^−2^ mol/L at pH 2 (RY84 solution, 50 mg/L) (Table 3, Section 2.7). In the initial pH range of pH 4–10 the ionic strength did not exceed 1 × 10^−3^ mol/L in any test series, so its effect on dye sorption was minor. Theoretically, a significant effect of ionic strength could be observed only at pH 2 (I = ~1.0–1.1 × 10^−2^ mol/L) and at pH 3 and pH (I = ~1.1–1.6 × 10^−2^ mol/L). Under these conditions, increased ionic strength could shorten the range of electrostatic interactions between the ionized functional groups of the sorbent and dyes due to shielding. Higher concentrations of background ions, such as Cl^−^ at pH 2–3 or Na^+^ at pH 11 (pH adjustments were made using HCl and NaOH), could limit the availability of sorption sites and reduce the electrostatic potential of the surface. For example, at pH 2–3, Cl^−^ ions could compete with anionic dyes for active, positively charged sites, while at pH 11, Na^+^ ions could compete with cationic dyes for negatively charged sorption sites. However, in this case (Figure 4a), no negative effect of increased ionic strength at pH 2 on the sorption of anionic dyes RB5 and RY84 was observed. In the test series with BV10 (cationic colorant), the lowest sorption effectiveness was noted at pH 11 (Figure 4b), but very similar efficiency was also seen at pH 9–10, where the ionic strength is much lower. Therefore, it can be concluded that ionic strength had a relatively small effect on dye sorption on the tested sorbent. This can be explained by the fact that potato peel has a heterogeneous and chemically complex structure, with some surface charges inaccessible to ions in solution, and electrostatic interactions occurring mainly at a local level. In such heterogeneous materials, the ionic shielding mechanism is less effective compared to sorbents with a uniform surface. In summary, for dye sorption on PP, changes in the sorbent surface charge caused by changes in solution pH, rather than changes in background ion concentration, play a key role.

**Figure 5:**
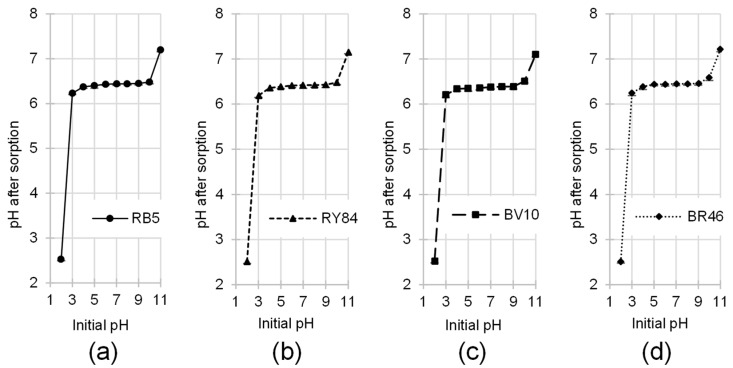
The dynamic transformation of the pH of solutions during the interaction between the dye: (**a**) RB5, (**b**) RY84, (**c**) BV10, (**d**) BR46 and the PP (average + standard deviation). Initial conc. of dyes = 50 mg/L. Temp. 25 °C.

The PP biomass exerted a influence on the alteration of the dye solution’s pH throughout the sorption process (Figure 5). Irrespective of the specific colorant present for initial pH values ranging from 3.0 to 10.0, the final pH consistently converged to a range between 6.2 and 6.5.

pH changes in solutions resulting from sorption are commonly observed during most sorption processes. At low pH, where the concentration of hydronium ions is high, protons from these ions bind to the functional groups present through protonation. Additionally, some hydronium ions may be adsorbed onto the sorbent surface. Both processes lead to an increase in the solution pH. Conversely, at high pH, where the concentration of hydroxide ions is higher, some protons leave the sorbent structure due to deprotonation of the functional groups. The released protons then combine with hydroxide ions to form water molecules, resulting in a decrease in the initial pH. The proportion of proton-donating to proton-accepting sites on the PP surface is the primary determinant of the final solution acidity. In practice, the system displays a strong tendency to self-regulate, bringing the pH to a value that closely approximates the characteristic pH_PZC_ of the potato peel biomass. As established in the previous section, the pH_PZC_ of PP is pH 6.43, which explains the observed pH stabilization in the range of pH 6.2–6.5 within the system.

### 3.3. Kinetics of Dyes Sorption onto PP

**Figure 6:**
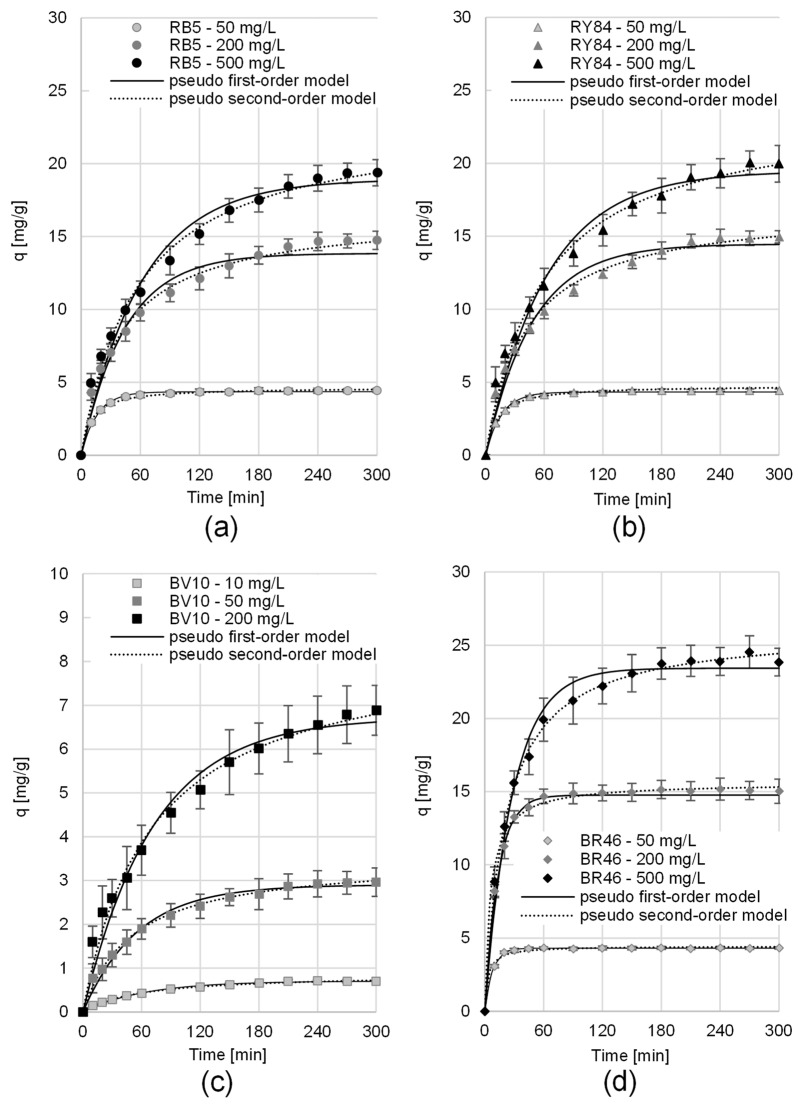
Sorption kinetics of: (**a**) RB5, (**b**) RY84, (**c**) BV10, (**d**) BR46 on PP (average + standard deviation). Pseudo-first-order model and pseudo-second-order model. Temp. 25 °C.

The sorption equilibrium time for RB5 and RY84 onto PP depended on the initial dye concentration and ranged from 150 min (at C_0_ = 50 mg/L) to 270 min (at C_0_ = 500 mg/L) (Table 4). The sorption intensity of these dyes onto potato peels was highest during the initial minutes of the reaction. The initial phase of the process was notably rapid; within the first 10 min, the adsorption capacity reached 30.8–50.0% of the equilibrium uptake (q_e_) for RB5, and 32.7–40.3% for RY84 (Figure 6).

Similar maximum sorption equilibrium times are well-documented in research on RB5 sorption onto buckwheat husks (up to 300 min) [73] and activated carbon from oil palm shells (up to 300 min) [74]. For RY84, comparable maximum equilibrium times were noted in research on the sorption of this dye onto aminated cotton fibers (up to 240 min) [63] and sunflower seed husks (240 min) [64].

The equilibration period for cationic species was found to be a function of the initial dye loading. Specifically, for BV10, the time required to reach a steady state shifted from 210 to 270 min as the concentration increased from 10 to 200 mg/L. A similar trend was noted for BR46, where the sorption duration extended from 45 min at 50 mg/L to 210 min at the highest concentration (Table 4). Comparable to the anionic colorants, the cationic dye capture was characterized by high initial rates; during the first 10 min window, the PP biomass sequestered 21.4–25.1% of q_e_ for BV10 and 37.9–70.9% for BR46.

Similar maximum times to reach sorption equilibrium have been reported in research on the removal of BV10 using biosorbents such as spent green tea leaves (240 min) [72]. For BR46, comparable sorption completion times were noted in experiments on the sorption of this dye onto rapeseed husks (180 min) [71], and cardboard waste (210 min) [75].

For every dye, the time required to achieve sorption equilibrium increased with the initial concentration of the dye (Table 4). The adsorption of dye molecules is primarily propelled by the chemical potential difference (concentration gradient) between the bulk liquid and the solid phase [76]. A more pronounced gradient facilitates the migration of sorbate species into the internal porous network of the PP; however, this increased penetration typically results in a prolonged duration to reach a steady state.

The shorter contact time observed for BR46 might be a consequence of its inferior molecular weight. Such compact molecular structures typically exhibit superior mobility, enabling more efficient navigation toward the active centers sequestered within the PP structure.

The time-dependent sorption data were interpreted through the lens of classic kinetic models, specifically the pseudo-first and pseudo-second-order frameworks. The resulting parameters and visual fits are detailed in Table 4 and Figure 6, respectively.

The statistical fit of the models to the experimental data was assessed by comparing the coefficients of determination (R^2^), where values closer to 1.0 indicate a better fit, as well as the Root Mean Square Error (RMSE) and the Akaike Information Criterion (AIC), where lower values indicate a better fit. Analysis of the R^2^, RMSE and AIC values calculated from the models shows that, in every research series, regardless of dye type or initial concentration, the pseudo-second-order model provided the best fit to the data. This is typical for the sorption of organic dyes onto sorbents based on plant biomass. The correlation between C_0_ and the resulting parameters (k_2_ and q_e_) was highly pronounced. Such concentration-dependent behavior is typically indicative of a moderate-to-low affinity of the dye molecules toward the sorbent’s functional groups.

**Figure 7:**
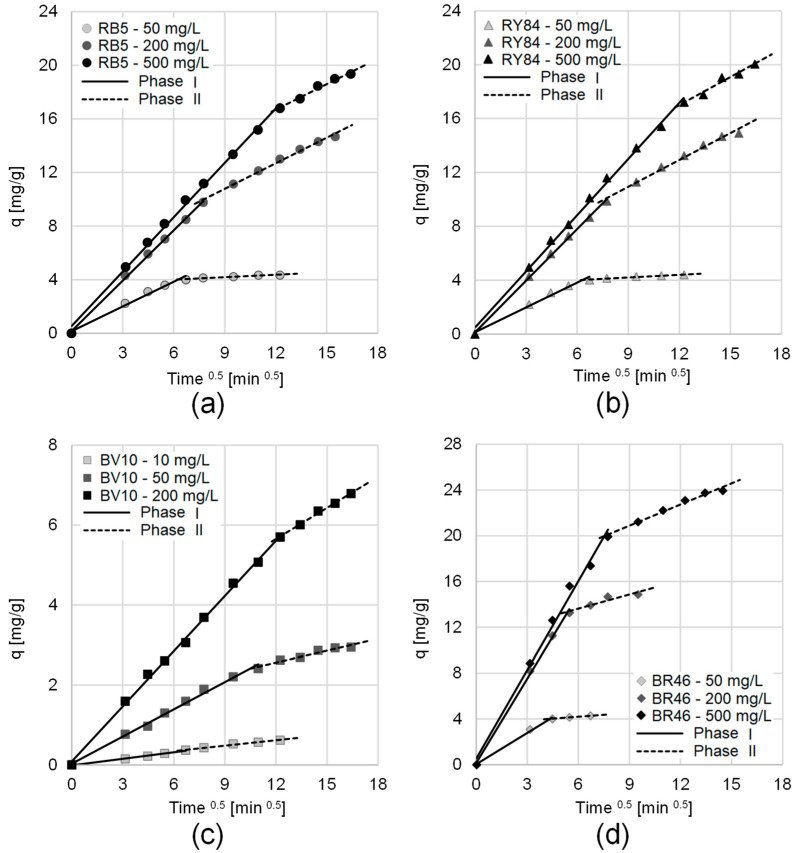
Intraparticle diffusion model for sorption of: (**a**) RB5, (**b**) RY84, (**c**) BV10, (**d**) BR46 on PP (calculations based on average values—from experimental data presented in Figure 6). Temp. 25 °C.

Mathematical modeling of the mass transfer resistance was performed using the intraparticle diffusion formalism, allowing for a deeper analysis of the experimental points (summarized in Table 5 and illustrated in Figure 7).

Analysis using this model showed that, across all experimental series, the dye sorption process onto PP occurred in two distinct phases. The initial phase had high sorption intensity. In this initial stage, dye underwent swift migration from the bulk solution toward the adsorbent’s external boundary. This resulted in the immediate saturation of the highly available functional groups situated on the outermost surface of the PP. The subsequent phase began once most active centers on the sorbent surface were saturated. This second stage was characterized by a greatly reduced availability of free active sites. As a result, the process entered a stage where dye molecules faced increased competition for the dwindling supply of vacant functional groups. This shift necessitated the penetration of the sorbate into the more sequestered internal pores of the PP, where mass transfer resistance is naturally higher and accessibility is limited. Therefore, this phase showed significantly lower sorption effectiveness compared to the first phase.

An upward trend in q_e(cal)_, k_d1_, and k_d2_ values was observed alongside increasing initial dye loadings for all systems studied (Table 4 and Table 5). These correlations suggest that the process efficiency is positively influenced by the starting concentration, primarily due to the enhanced chemical potential gradient. This intensified differential acts as the fundamental propulsion mechanism, accelerating the transfer of dye ions from the aqueous phase to the PP surface.

As illustrated in Figure 7, the initial linear segment of the plots for all series intersects the origin of the coordinates. This implies that the external mass transfer resistance (film diffusion) is negligible, indicating that intra-particle diffusion acts as the primary rate-controlling mechanism during the dye uptake process [77].

### 3.4. Maximum Sorption Capacity of PP

**Figure 8:**
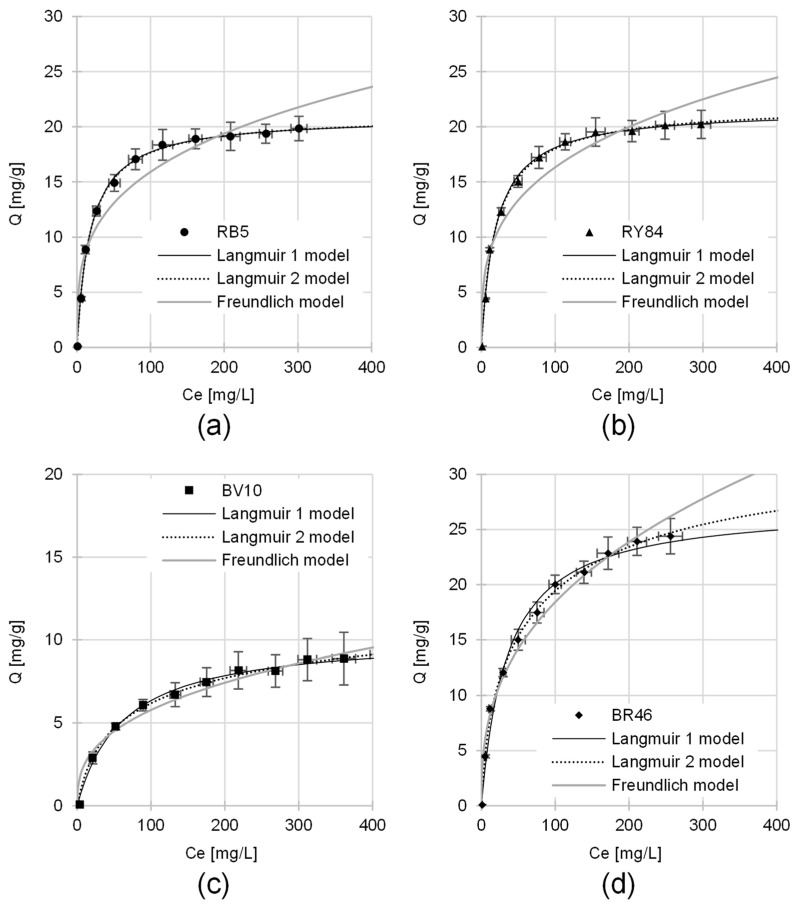
Isotherm of sorption of: (**a**) RB5, (**b**) RY84, (**c**) BV10, (**d**) BR46 on PP (average + standard deviation). Langmuir 1, Langmuir 2, and Freundlich models. Temp. 25 °C.

To evaluate the equilibrium characteristics and determine the maximal uptake potential of the PP sorbent, the experimental results were fitted to three well-established isotherms: the Langmuir (types 1 and 2) and the Freundlich equations (detailed in Table 6 and Figure 8). The statistical fit of the models to the experimental data was assessed by comparing the R^2^, RMSE and AIC. Across all experimental conditions, statistical indicators (R^2^, RMSE, and AIC) consistently pointed to the superiority of the Langmuir (1 and 2) models over the Freundlich equation (Table 6). This strongly suggests a specific dye sorption mechanism in which only one dye molecule (or ion) can bind to a single sorption site on the sorbent surface. Consequently, the dyes adsorbed onto PP form a monolayer, allowing for the exchange of active sites [78]. For a dye-saturated sorbent, equimolar exchange is also possible between the dyes present in the monolayer and those in the solution.

The goodness-of-fit metrics R^2^ and RMSE) revealed that the Langmuir 2 equation generally outperformed Langmuir 1 in terms of mathematical accuracy. The lower AIC values observed for the Langmuir 1 model in some cases are a direct result of the “principle of parsimony”, as the AIC favors models with fewer estimated parameters. The superiority of the Langmuir 2 linearization (evidenced by R^2^ and RMSE metrics) implies that the sorptive surface of the potato peels is not strictly uniform. Instead, it suggests the involvement of at least two distinct classes of binding sites, each contributing significantly to the sequestration of dye molecules [79]. However, the very high standard errors for the parameters b_1_ and b_2_ in the Langmuir 2 model (standard error > 50% of the parameter value; see Table 6) indicate a lack of statistical significance for these estimated parameters, leading to very low model reliability. Therefore, the parameters determined from the Langmuir 1 model will be used for further data description.

As noted in Section 3.2, the key active sites for the anionic dyes RB5 and RY84, as well as the cationic dye BV10, are likely the protonated functional groups (amino and hydroxyl) on the PP surface. However, the role of hydronium ions adsorbed onto PP as potential sorption sites for these dyes cannot be ruled out. In contrast, ionized (deprotonated) carboxyl groups (-COO-) probably play an vital role in the binding of BR46. Additionally, the sorption of RB5, RY84, BV10, and BR46 onto PP may also occur through π-π interactions associated with the lignin’s benzene rings (a component of PP) and the aromatic rings of the dyes.

The theoretical maximum sorption capacity of the PP biomass was determined to be 20.85 mg/g and 21.63 mg/g for RB5 and RY84, respectively. In the case of cationic colorants, the PP sorbent showed a sequestration capacity of 10.28 mg/g (BV10) and 27.15 mg/g (BR46) (Table 6, Langmuir 1).

The values of the constant K_C_ (Langmuir model), which indicate the affinity of the sorbates for the active sites, are relatively low (<0.1) for the tested dyes (Table 6). This suggests that PP achieves full sorption efficiency only at very high dye concentrations in solutions.

The sorption capacities of potato peels for RB5, RY84, and BR46 are at a similar level. The effective sorption of both types of dyes makes PP a versatile sorbent. The lower sorption of BV10 compared to BR46 is presumably due to Basic Violet 10 possessing a carboxyl functional group, which is atypical for a cationic dye, as explained in Section 3.2.

Table 7 and Table 8 compare the sorption properties of PP with those of other agro-waste sorbents (literature data).

As shown in Table 7, PP stands out as a highly proficient adsorbent for anionic dyes, surpassing many plant-derived materials such as cereal husks or cultivated plant biomass. Remarkably, the sorption metrics for RB5 on PP are on par with various activated carbon variants (Table 7).

The sorption capacity of PP for BR46 is superior to that of sawdust or cardboard-based sorbents (Table 8). For both cationic dyes, better waste-based sorbents include cultivated seed husks (e.g., rapeseed) or spent green tea leaves, which is presumably related to the higher acidity of these sorbents. Furthermore, the sorption of these dyes is several times less effective than with activated carbons (Table 8).

Although potato peels do not match the sorption capacities of commercial activated carbons, they represent a promising alternative due to their abundance and cost-effectiveness. Moreover, PP can be considered a universal sorbent because, unlike most sorbents based on unmodified plant biomass, it demonstrates similar sorption effectiveness for both anionic and cationic colorants.

It is important to address the need for wastewater pH correction to achieve both effective sorption and post-process pH neutralization. The PP sorption capacities for RB5, RY84, and BV10 determined in this study were obtained at an initial solution pH of 2.0. Since colored wastewater, particularly from the textile industry, typically has a higher pH (6–10) [91], adjusting it to pH 2.0 would require a costly pre-acidification step involving the addition of strong acids. After sorption with PP (at a dosage of 10 g/L), the treated wastewater would have a pH of approximately 2.5 and would need to be neutralized to pH 6.5–8.5 with alkali before discharge to the sewer system or the environment [92]. The need for double pH adjustment can significantly increase operating costs due to chemical consumption. Additionally, operating infrastructure and process equipment under highly acidic conditions (pH 2.0–2.5) would require specialized, acid-resistant construction materials, increasing capital expenditure and maintenance costs. One way to address this issue is through process integration, such as replacing strong bases with an in-house alkaline wastewater stream [93], or performing sorption on PP at higher pH values (e.g., 4.0–5.0), even if this results in lower efficiency. Ultimately, the cost-effectiveness of using PP as a sorbent for treating real colored wastewater requires detailed economic and technical analysis in future studies.

The fate of the PP biomass following the sorption process is a critical aspect to consider. Due to the low physical durability of potato peels, their reuse is challenging. Additionally, attempts to regenerate these biosorbents through sorption and desorption cycles typically require costly chemical reagents, such as concentrated acids or bases [94]. This process also produces highly concentrated desorption wastewater, which requires complex and expensive treatment. Economically, the total operational cost of regeneration and subsequent wastewater treatment often exceeds the cost of obtaining new, low-cost PP. Therefore, given the high availability and low price of the raw material, PP should primarily be considered a single-use biosorbent.

The main environmental concern regarding dye-loaded PP is the risk of leaching concentrated, potentially toxic, or persistent dye molecules if the spent material is improperly landfilled. To mitigate this risk, methods focusing on energy or material recovery are essential.

One key option is energy recovery. The components of PP and the adsorbed dyes have a high calorific value, making co-incineration in a combined heat and power plant an effective disposal strategy. This process offers the advantage of complete destruction of the dye structure at high temperatures, effectively eliminating the risk of pollutant persistence. However, careful attention must be paid to flue gas monitoring, especially when dealing with dyes containing heteroatoms such as metals, chlorine, or sulfur, to prevent the emission of toxic combustion products like NO_x_ or heavy metal oxides [95].

Alternatively, spent PP can be directed to anaerobic digestion for biogas production. Research indicates that the presence of organic dyes, such as Basic Red 46, in the fermentation feedstock does not exert a significant inhibitory effect on methanogenesis, even at elevated concentrations [96]. Nevertheless, this option requires vigilance, as any heavy metals present in complex dyes may accumulate in the resulting digestate, potentially limiting its safe use as an agricultural soil amendment.

The most advanced solution for material valorization is the thermal conversion of spent PP into activated carbon. The carbonization and chemical or physical activation process uses high temperatures, which not only stabilizes the material but also leads to the irreversible degradation of the adsorbed organic contaminants. The resulting activated carbon, in powder or pellet form, is a high-value product that can be used as a fully functional adsorbent in downstream industrial wastewater treatment, effectively closing the material loop [97].

## 4. Conclusions

Potato peels are an inexpensive and widely available material that can be used as an unconventional sorbent for removing both cationic and anionic dyes. The sorption capacities of potato peels (PP) were 10.28 mg/g for BV10, 27.15 mg/g for BR46, 20.85 mg/g for RB5, and 21.15 mg/g for RY84. PP binds anionic dyes more effectively than most plant biomass-based sorbents. For anionic dyes and BV10, the most important sorption centers were likely protonated functional groups (-NH_3_^+^, -OH_2_^+^). For BR46, however, the primary active sites were ionized carboxyl groups.

The solution pH acts as a decisive factor in the sorption of dyes on potato peels. Optimal uptake for the anionic species (RB5, RY84) and the cationic BV10 was observed at pH 2, whereas BR46 exhibited peak affinity at pH 6. The point of zero charge pH_PZC_ of the PP matrix was identified at 6.43, confirming the prevalence of acidic functional groups, such as carboxyls, which impart a net negative charge to the surface under near-neutral conditions.

The time required to reach sorption equilibrium on PP increased with the initial dye concentration and ranged from 180 to 270 min for RB5, RY84, and BV10, and from 45 to 210 min for BR46. Sorption of each dye onto PP occurred in two distinct phases. The kinetic parameters derived from the intraparticle diffusion framework suggest that external mass transfer resistance (film diffusion) played a negligible role in the overall process. This indicates that the rate-determining step was primarily governed by the migration of dye species within the porous architecture of the PP.

## Figures and Tables

**Table 1:** Constituent analysis of potato peels [36,42,43,44].

Compound	Content (Range) [%]
Starch	23.0–52.1
Cellulose	32.4–55.0
Hemicellulose	10.0–12.0
Monosaccharides (soluble)	1.0–7.3
Lignin	14.0–20.0
Proteins	8.0–14.2
Lipids	1.0–2.6
Ash and minerals	2.1–9.1

**Table 2:** Data summary for the tested dyes.

Dye Name	Reactive Black 5 (RB5)	Reactive Yellow 84 (RY84)	Basic Violet 10 (BV10)	Basic Red 46 (BR46)
Other trade names	Diamira Black B, Begazol Black B, Remazol Black B	Lamafix Yellow HER, Apollocion Yellow H-E4R, Active Yellow HE-4R	Basic Red RB, Violet B, Rhodamine B,	Sevron Fast Red GRL, Cationic Red X-GRL, Anilan Red GRL
Chemical formula	C_26_H_21_N_5_Na_4_O_19_S_6_	C_56_H_38_Cl_2_N_14_Na_6_O_20_S_6_	C_28_H_31_ClN_2_O_3_	C_18_H_21_BrN_6_
Molecular weight	991.8 g/mol	1628.2 g/mol	479.0 g/mol	321.4 g/mol
Dye class	double azo dye	double azo dye	xanthene dye	single azo dye
Dye type	anionic (reactive)	anionic (reactive)	cationic	cationic
λ_max_	600 nm	356 nm	554 nm	530 nm
Uses	dyeing wool and cotton	dyeing polyester, cotton, rayon	dyeing textiles, paper, leather	dyeing leather, paper, wool

**Table 3:** Computational ionic strength of dye solutions (conc. 50 mg/L) as a function of pH.

Dye	Ionic strength I [mol/L] of dye solutions (50 mg/L) at different pH				
	pH 2	pH 3	pH 4	pH 5	pH 6
RB5	1.05 × 10^−2^	1.50 × 10^−3^	6.04 × 10^−4^	5.14 × 10^−4^	5.05 × 10^−4^
RY84	1.06 × 10^−2^	1.62 × 10^−3^	7.24 × 10^−4^	6.34 × 10^−4^	6.25 × 10^−4^
BV10	1.02 × 10^−2^	1.16 × 10^−3^	2.56 × 10^−4^	1.66 × 10^−4^	1.57 × 10^−4^
BR46	1.01 × 10^−2^	1.10 × 10^−3^	2.04 × 10^−4^	1.14 × 10^−4^	1.05 × 10^−4^
Dye	Ionic strength I [mol/L] of dye solutions (50 mg/L) at different pH				
	pH 7	pH 8	pH 9	pH 10	pH 11
RB5	5.04 × 10^−4^	5.05 × 10^−4^	5.14 × 10^−4^	6.04 × 10^−4^	1.50 × 10^−3^
RY84	6.24 × 10^−4^	6.25 × 10^−4^	6.34 × 10^−4^	7.24 × 10^−4^	1.62 × 10^−3^
BV10	1.56 × 10^−4^	1.57 × 10^−4^	1.66 × 10^−4^	2.56 × 10^−4^	1.16 × 10^−3^
BR46	1.04 × 10^−4^	1.05 × 10^−4^	1.14 × 10^−4^	2.04 × 10^−4^	1.10 × 10^−3^

**Table 4:** Kinetic parameters of anionic and cationic dye sorption on PP determined by the pseudo-first-order and pseudo-second-order models. Experimental values are presented as mean ± standard deviation. Model parameters are presented as estimate ± standard error.

Dye	Dye Conc.	Pseudo-First-Order Model		Pseudo-Second-Order Model		Exp. Data	Equil. Time
		k_1_	q_e,(cal.)_	k_2_	q_e,(cal.)_	q_e,exp_	
	[mg/L]	[1/min]	[mg/g]	[g/mg*min]	[mg/g]	[mg/g]	[min]
RB5	50	0.0631 ± 0.0041	4.35 ± 0.04	0.0225 ± 0.0016	4.65 ± 0.06	4.42 ± 0.21	180
	200	0.0224 ± 0.0025	13.84 ± 0.45	0.0015 ± 0.0002	16.61 ± 0.45	14.66 ± 0.63	240
	500	0.0162 ± 0.0017	18.93 ± 0.60	0.0008 ± 0.0001	23.02 ± 0.71	19.35 ± 0.69	270
RY84	50	0.0635 ± 0.0027	4.32 ± 0.04	0.0197 ± 0.0015	4.78 ± 0.06	4.43 ± 0.10	180
	200	0.0209 ± 0.0022	14.47 ± 0.43	0.0015 ± 0.0002	17.02 ± 0.41	14.91 ± 0.58	240
	500	0.0160 ± 0.0016	19.47 ± 0.61	0.0007 ± 0.0001	23.75 ± 0.71	20.04 ± 0.80	270
BV10	10	0.0160 ± 0.0013	0.71 ± 0.02	0.0193 ± 0.0014	0.87 ± 0.02	0.71 ± 0.04	210
	50	0.0179 ± 0.0014	2.90 ± 0.07	0.0057 ± 0.0004	3.50 ± 0.07	2.95 ± 0.26	270
	200	0.0139 ± 0.0015	6.73 ± 0.24	0.0017 ± 0.0003	8.39 ± 0.32	6.79 ± 0.66	270
BR46	50	0.1268 ± 0.0049	4.30 ± 0.04	0.0683 ± 0.0085	4.44 ± 0.20	4.28 ± 0.10	45
	200	0.0730 ± 0.0033	14.76 ± 0.16	0.0110 ± 0.0007	15.61 ± 0.38	14.88 ± 0.71	90
	500	0.0354 ± 0.0028	23.44 ± 0.45	0.0019 ± 0.0001	26.10 ± 0.23	23.94 ± 1.06	210
Model Evaluation Metrics							
Dye	Dye Conc.	Pseudo-First-Order Model			Pseudo-Second-Order Model		
	[mg/L]	R^2^	RMSE	AIC	R^2^	RMSE	AIC
RB5	50	0.9948	0.1479	−34.23	0.9955	0.0875	−44.72
	200	0.9727	0.6305	−9.33	0.9919	0.2452	−30.05
	500	0.9760	0.9740	3.33	0.9908	0.4640	−16.01
RY84	50	0.9963	0.1428	−29.92	0.9964	0.0921	−37.28
	200	0.9754	0.5049	−11.07	0.9938	0.2760	−23.88
	500	0.9769	1.0556	5.31	0.9914	0.6130	−7.75
BV10	10	0.9921	0.0404	−60.15	0.9971	0.0276	−67.80
	50	0.9864	0.1316	−41.30	0.9958	0.0625	−55.58
	200	0.9769	0.3807	−19.15	0.9893	0.2370	−30.62
BR46	50	0.9945	0.1922	−14.07	0.9952	0.0917	−8.92
	200	0.9948	0.1479	−34.23	0.9955	0.0875	−44.72
	500	0.9727	0.6305	−9.33	0.9919	0.2452	−30.05

**Table 5:** Dye translocation rates within the sorbent structure, quantified through intraparticle diffusion modeling (calculations based on average values—from experimental data presented in Figure 6).

Dye	Dye Conc.	Phase I			Phase II		
		k_d1_	Duration	R^2^	k_d2_	Duration	R^2^
	[mg/L]	[mg/(g*min^0.5^)]	[min]	-	[mg/(g*min^0.5^)]	[min]	-
RB5	50	0.6142	45	0.9846	0.0575	135	0.9524
	200	1.2535	60	0.9984	0.6339	180	0.9917
	500	1.3533	150	0.9970	0.6384	120	0.9836
RY84	50	0.6147	45	0.9865	0.0600	135	0.9463
	200	1.2782	60	0.9986	0.6656	180	0.9876
	500	1.3912	150	0.9969	0.6913	120	0.9711
BV10	10	0.0545	90	0.9918	0.0448	120	0.9872
	50	0.2270	120	0.9931	0.0992	150	0.9553
	200	0.4607	150	0.9980	0.2596	120	0.9956
BR46	50	0.9134	20	0.9965	0.1134	45	0.9951
	200	2.4494	30	0.9978	0.4114	60	0.8992
	500	2.5834	60	0.9920	0.6171	150	0.9814

**Table 6:** Mathematical constants obtained from the application of Langmuir (types 1 and 2) and Freundlich adsorption models. Model parameters are presented as estimate ± standard error.

Dye	Langmuir 1 Model				Freundlich Model				
	Q_max_		K_c_		k		n		
	[mg/g]		[L/mg]		-		-		
RB5	20.85 ± 0.33		0.0540 ± 0.0042		4.177 ± 0.935		3.450 ± 0.045		
RY84	21.63 ± 0.34		0.0514 ± 0.0038		4.15 ± 0.902		3.369 ± 5.630		
BV10	10.28 ± 0.24		0.0162 ± 0.0014		1.098 ± 0.227		2.772 ± 0.313		
BR46	27.15 ± 0.874		0.0285 ± 0.0034		3.286 ± 0.518		2.671 ± 0.228		
Dye	Langmuir 2 Model								
	Q_max_		b_1_		K_1_		b_2_		K_2_
	[mg/g]		[mg/g]		[L/mg]		[mg/g]		[L/mg]
RB5	20.91 ± 23,977		20.46 ± 16,954		0.0547 ± 1031		0.44 ± 16,954		0.0547 ± 47,651
RY84	21.90 ± 45.39		19.75 ± 33.02		0.0567 ± 0.0627		2.15 ± 31.15		0.0139 ± 0.1992
BV10	12.01 ± 23.64		6.34 ± 23.28		0.0037 ± 0.0115		5.67 ± 4.09		0.0370 ± 0.0115
BR46	31.88 ± 8.13		20.27 ± 4.19		0.0079 ± 0.0685		11.60 ± 6.97		0.0953 ± 0.0684
Model Evaluation Metrics									
Dye	Langmuir 1 Model			Langmuir 2 Model			Freundlich Model		
	R^2^	RMSE	AIC	R^2^	RMSE	AIC	R^2^	RMSE	AIC
RB5	0.9940	0.4795	−12.22	0.9940	0.4795	−12.22	0.9029	2.0104	19.33
RY84	0.9966	0.5970	−0.12	0.9969	0.5510	2.21	0.9017	2.1120	21.41
BV10	0.9927	0.3950	−6.23	0.9971	0.3750	−5.45	0.9120	0.7780	6.94
BR46	0.9889	1.1890	8.61	0.9977	0.8400	2.21	0.9767	1.3460	11.02

**Table 7:** Comparison of the sorption properties of various sorbents towards RB5 and RY84 dyes.

Dye	Sorbent	Q_max_ [mg/g]	pH of Sorption	Time of Sorption [min]	Source
RB5	Activated carbon (powder)	58.8	-	-	[80]
	Activated carbon from palm shells	25.1	2	300	[74]
	Potato Peels	20.9	2	270	This work
	Activated carbon from wood (walnut)	19.3	5	400	[81]
	Wheat straw	15.7	3	195	[82]
	Rapeseed husks	15.2	3	180	[33]
	*Eriobotrya japonica* seed husks	13.8	3	150	[83]
	Cotton seed husks	12.9	2	30	[62]
RY84	Activated carbon from the *Borassus flabellifer* plant	40.0	-	-	[84]
	Potato Peels	21.6	2	270	This work
	Cotton fibers	15.9	2	240	[63]
	Rapeseed husks	13.7	3	180	[33]
	Wool	11.0	7	180	[85]

**Table 8:** Comparison of the sorption properties of various sorbents towards BR46 and BV10 dyes.

Dye	Sorbent	Q_max_ [mg/g]	pH of Sorption	Time of Sorption [min]	Source
BV10	Commercial active carbon powder	72.5	4	1440	[28]
	Activated carbon (palm shell-based)	30.0	3	-	[86]
	Spent green tea leaves	26.7	3	240	[72]
	Corrugated cardboard (used)	24.7	2	210	[75]
	Rapeseed husks	20.9	3	180	[71]
	Sugar cane fiber	10.4	-	-	[87]
	Potato Peels	10.3	2	270	This work
BR46	Activated carbon “Chemviron”	106.0	7.4	120	[88]
	*Cerbera odollam* biomass activated carbon	65.7	7	90	[89]
	Rapeseed husks	59.1	6	180	[71]
	Spent green tea leaves	58.0	6	240	[72]
	Potato Peels	27.2	6	210	This work
	Office paper (used)	19.6	6	90	[75]
	Wood sawdust	19.2	-	120	[90]

## Data Availability

The original contributions presented in this study are included in the article. Further inquiries can be directed to the corresponding author.
